# Essential histidine residues in Bombyx mori nucleopolyhedrovirus GP64 mediate pH-dependent membrane fusion

**DOI:** 10.1128/spectrum.00876-25

**Published:** 2025-07-15

**Authors:** Haijue Tian, Kai Chen, Xinyu Li, Ying Xu, Bifang Hao, Jinshan Huang

**Affiliations:** 1 Jiangsu Key Laboratory of Sericultural and Animal Biotechnology, School of Biotechnology, Jiangsu University of Science and Technology 12676, Zhenjiang, China; 2 Key Laboratory of Silkworm and Mulberry Genetic Improvement, Ministry of Agriculture and Rural Affairs, Sericultural Scientific Research Center, Chinese Academy of Agricultural Sciences, Zhenjiang, China; Barnard College, Columbia University, New York, New York, USA

**Keywords:** baculovirus, membrane fusion protein, GP64, BmNPV, pH sensor

## Abstract

Enveloped viruses initiate host cell entry through membrane fusion mediated by viral fusion proteins. In group I alphabaculoviruses, the class III membrane fusion protein GP64 mediates virus–cell fusion under acidic conditions. In Autographa californica multicapsid nucleopolyhedrovirus (AcMNPV), three key histidine residues in GP64 function as pH sensors. In contrast, Bombyx mori nucleopolyhedrovirus (BmNPV)—despite its high genomic similarity to AcMNPV—exhibits a lower fusion pH threshold and retains its signal peptide in host cells, introducing additional histidine residues absent in AcMNPV GP64. To elucidate the role of these histidines, we generated 14 single-histidine-to-alanine mutants of BmNPV GP64 and evaluated their expression, membrane localization, and fusogenicity in BmN cells. Although all mutants correctly formed trimers and localized to the cell surface, several mutations either abolished or diminished low-pH-induced syncytia formation and altered the binding profile of the conformation-sensitive AcV1 antibody. Moreover, the reintroduction of these mutants into a *gp64*-null bacmid revealed that specific residues (H168, H172, H342, and H351) are essential for pH-induced conformational transitions and viral infectivity, thereby underscoring their role as pH sensors. These results not only enhance our understanding of the molecular basis underlying BmNPV GP64-mediated membrane fusion but also lay the foundation for developing targeted strategies to control BmNPV infections in economically important silkworms.

**IMPORTANCE:**

Understanding the molecular determinants of viral fusion is essential for elucidating virus-host interactions and adaptation. This study provides novel insights into how specific histidine residues in BmNPV GP64 govern pH-dependent conformational changes necessary for membrane fusion. By dissecting the contributions of these residues through mutagenesis and functional assays, our work clarifies differences in fusion activation between closely related alphabaculoviruses. Such insights are crucial not only for advancing our basic knowledge of viral entry mechanisms but also for informing the design of antiviral strategies that could interfere with viral fusion processes. The identification of critical pH-sensing residues in BmNPV GP64 lays a foundation for future structural studies and understanding pH-dependent activation in other enveloped viruses.

## OBSERVATION

**FIG 1:**
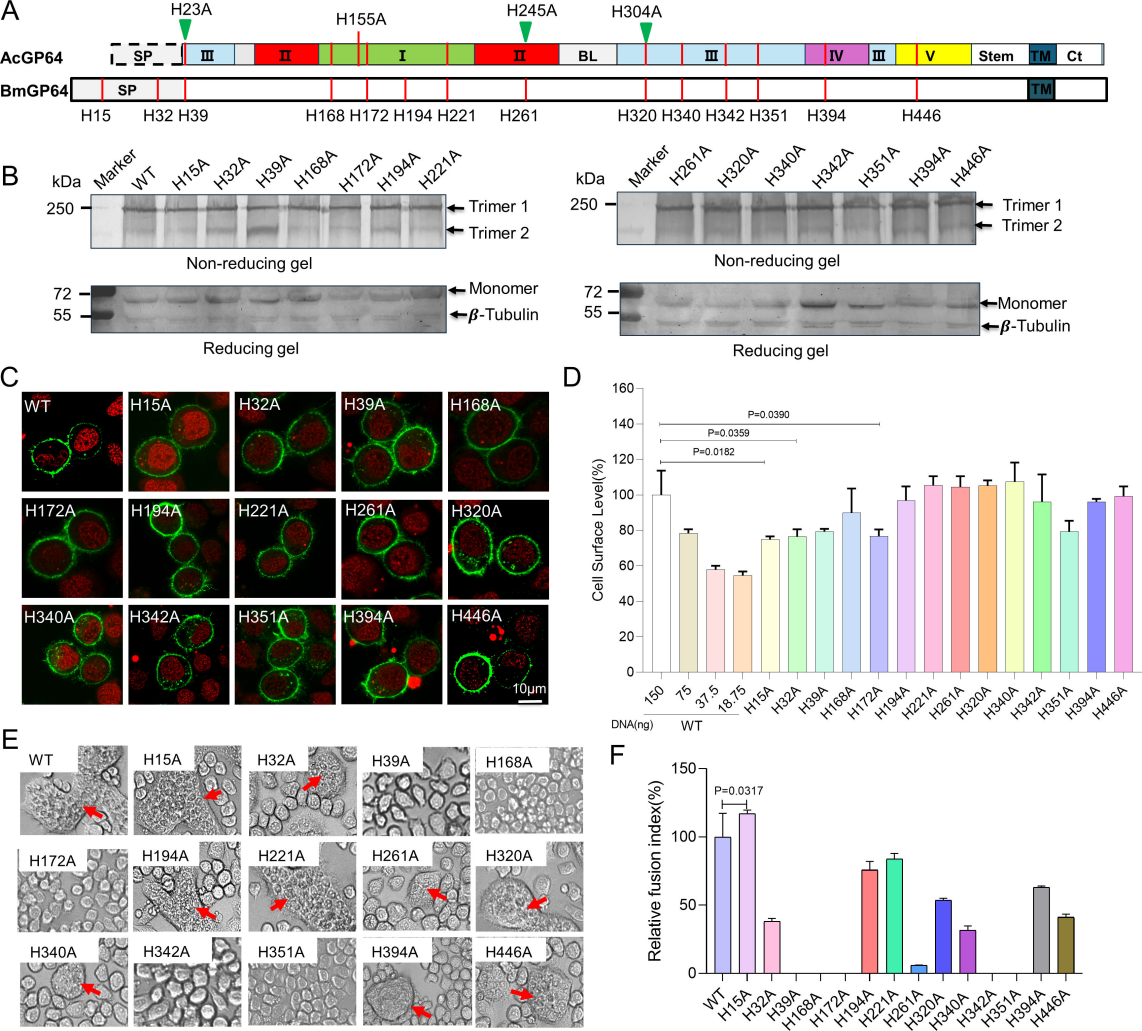
Impact of histidine mutations in BmNPV GP64 on expression and fusogenicity. (**A**) Schematic diagram of histidine mutations in GP64. The linear domain represents domains I-V in the GP64 post-fusion structure. Red lines indicate histidine residues in GP64, while green triangles mark multiple-histidine sensing sites in AcMNPV GP64. The cleaved signal peptide (SP) of AcMNPV GP64 is enclosed in a gray dashed box, whereas the uncleaved SP of BmNPV GP64 is enclosed in a gray solid box. (**B**) Western blot analysis of GP64 expression and trimerization. BmN cells in a 12-well plate were transfected with 1500 ng of WT or mutant GP64 DNA. At 72 h post-transfection (h p.t.), cells were harvested for western blot under non-reducing (upper) and reducing (lower) conditions using GP64 and β-tubulin antibodies. (**C**) Immunofluorescence analysis of histidine mutants. BmN cells in confocal dishes (NEST Biotech, Wuxi, China) were transfected with 1500 ng of plasmid DNA and fixed at 72 h p.t. GP64 localization was detected using a GP64 primary antibody (AcV5, Santa Cruz, CA, USA) and a FITC-labeled secondary antibody. Nuclei were stained with RedDot1 (Biotium, CA, USA). Bar: 10 µm. (**D**) Cell surface expression levels of histidine mutants. The relative cell surface level of each histidine mutant was quantified by cELISA at 72 h p.t. using AcV5 antibody. BmN cells in a 96-well plate were transfected with 150 ng of the respective plasmid DNA. Values represent means from triplicate transfections, normalized to WT GP64 (representing 100%). A standard curve was generated by transfecting decreasing amounts of WT GP64-expressing plasmid (left panel), with plasmid DNA quantities indicated below the graph. (**E**) Syncytium formation assays. BmN cells in a 24-well plate were transfected with 750 ng of WT or mutant plasmid DNA. At 72 h p.t., syncytia were induced by a 10 min incubation in serum-free medium at pH 4.5, followed by a 4 h recovery in normal medium. Enlarged images highlight syncytia (arrowheads). (**F**) Relative fusion index of histidine mutants. Fusion activity was quantified as the percentage of nuclei within syncytia relative to the total nuclei in the field. The fusion index represents the ratio of fusion activity to cell surface expression, with WT GP64 set at 100%.

Enveloped viruses enter host cells through conformational rearrangements of membrane fusion proteins (MFPs), triggered by endosomal acidification, receptor binding, or proteolytic priming (1, 2). In group I alphabaculoviruses, the class III MFP GP64—acquired during evolution—mediates the fusion of viral and host membranes in response to low pH (3). Histidine residues have long been recognized as critical determinants in low-pH-triggered viral membrane fusion (4), and recent structure studies of the pre-fusion state have identified multiple histidines (Fig. 1A; H23, H245, and H304) in Autographa californica multicapsid nucleopolyhedrovirus (AcMNPV) GP64 that act as pH sensors (3, 5).

Bombyx mori nucleopolyhedrovirus (BmNPV), a close relative of AcMNPV with high genomic similarity but a distinct host range (6), exhibits a notable lower fusion pH threshold. While AcMNPV GP64 triggers fusion at approximately pH 5.0 (3), BmNPV GP64 requires a substantially lower pH (<4.5) (7, 8), suggesting a divergent pH-sensing mechanism. Our recent work reveals that, in host cells, BmNPV GP64 retains its signal peptide (SP)—in contrast to non-host cells—thereby influencing cell surface localization, cholesterol dependence, and its dual functions in membrane fusion and virus budding (9–11). Notably, the uncleaved SP of BmNPV GP64 contains two additional histidine residues compared to SP-cleaved AcMNPV GP64, raising the possibility that these extra histidines contribute to its unique pH-sensing properties.

To address this hypothesis, we generated 14 single-histidine mutants of BmNPV GP64 by substituting each histidine with alanine via overlapping PCR (Fig. 1A). These mutants were cloned into the pIZ/V5 vector (Thermo Fisher Scientific, USA) and transiently expressed in BmN cells. Western blot analyses under non-reducing conditions confirmed that none of these mutations disrupted GP64 trimerization (Fig. 1B), and immunofluorescence assays verified that all mutant proteins trafficked to the plasma membrane (Fig. 1C). However, mutations in the N-terminus histidines, specifically H15A, H32A, and H172A, resulted in significantly reduced surface expression as measured by cell-based ELISA (cELISA) (Fig. 1D). Upon low pH treatment to induce syncytia formation, distinct differences in fusogenicity emerged. Although syncytia formed in cells expressing H15A, H32A, H194A, H221A, H320A, H340A, H394A, and H446A mutants (Fig. 1E, arrowheads), mutations H39A, H168A, H172A, H342A, and H351A completely abolished fusogenicity. Quantitative analysis of the fusion index revealed that, while H15A even showed enhanced fusogenicity relative to wild-type (WT) GP64, the other mutations exhibited variable levels of impairment (Fig. 1F).

**FIG 2:**
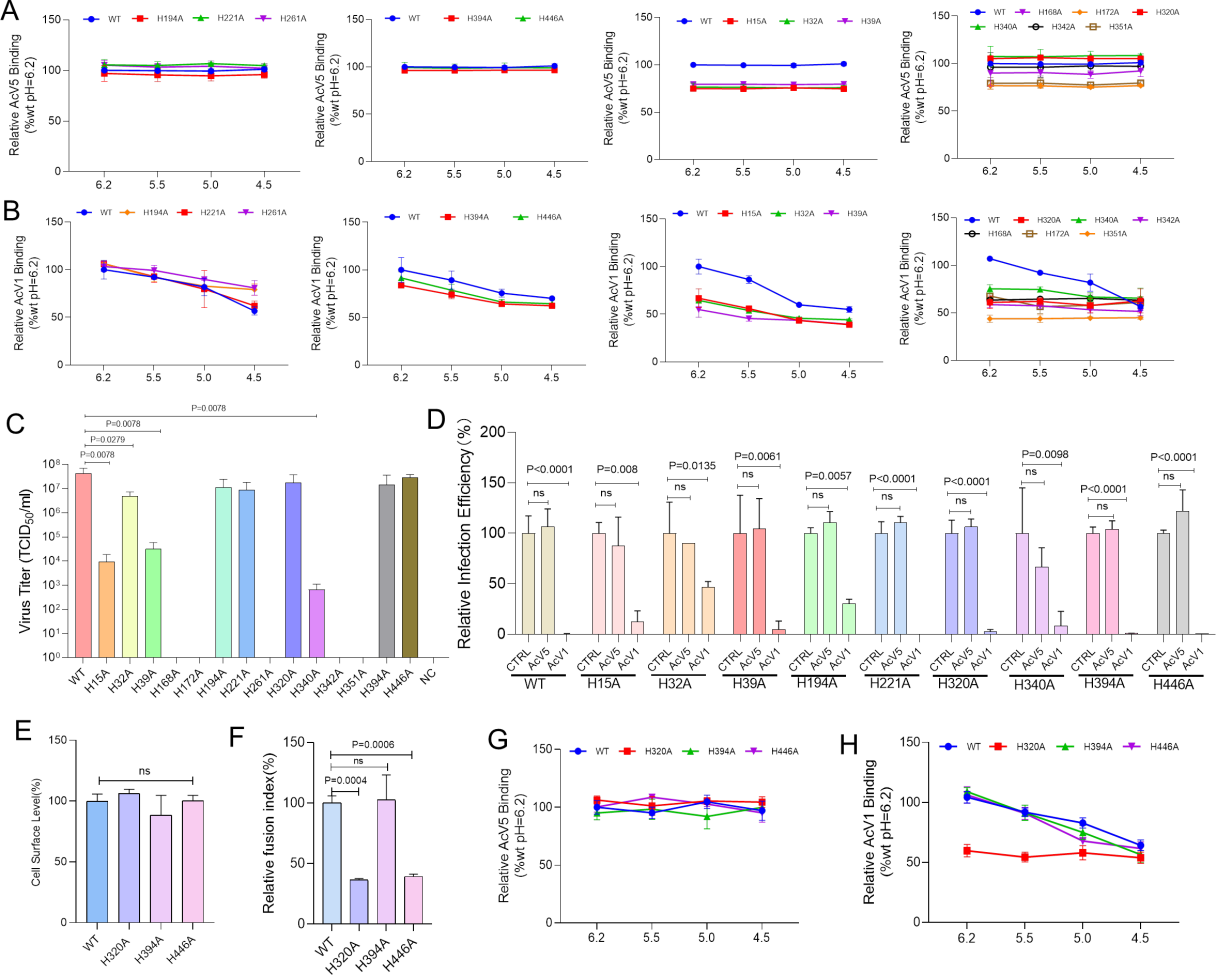
.Analysis of low pH-triggered conformational changes in histidine mutants. (**A, B**) Conformational changes in histidine mutants detected with AcV5 (**A**) and AcV1 (**B**) antibodies. BmN cells in a 96-well plate were transfected with 150 ng of WT or mutant plasmid DNA. At 48 h p.t., cells were incubated at the indicated pH values for 30 min, fixed with 4% paraformaldehyde, and subjected to a cELISA using AcV1 and AcV5 antibodies. Binding signals were normalized to WT GP64 at pH 6.2. Error bars represent the standard deviations (SD) from three independent replicates (*n *= 3). (**C**) Progeny BV production of mutants. Mutated gp64 genes were introduced into a gp64-null bacmid (BmBac^Δgp64^) via transposition. BmN cells in a 6-well plate were transfected with 3000 ng bacmid DNA using PEI, and BmBac^Δgp64^-egfp served as a negative control. BV was collected at 144 h p.t. for titration via the endpoint dilution assay method. (**D**) Neutralization assay of recombinant virus infection. WT and mutant viruses were pre-incubated with AcV5 or AcV1 antibodies (1:1500 dilution) for 2 h before infecting cells at MOI of 5 (MOI = 0.01 for H15A, H39A, and H340A). Infection rates were assessed by flow cytometry at 48 h p.i. Relative infection efficiency was calculated by normalizing the infection rate of antibody-treated samples to the untreated control. (**E**) Cell surface levels of mutant viruses. BmN cells were infected with WT, H320, H394A, and H446A viruses at an MOI of 1 TCID_50_. The relative cell surface level of each histidine mutant was measured by cELISA using AcV5 antibody at 48 h p.i. (**F**) Relative fusion index of histidine-mutated virus. Infected cells were incubated in serum-free medium at pH 4.5 to induce syncytia at 48 h p.i., followed by a 4 h recovery in normal medium. Fusion activity was quantified as the percentage of nuclei within syncytia relative to the total nuclei in the field. The fusion index represents the ratio of the fusion activity to cell surface expression. (**G, H**) Conformational changes in virus-infected cells detected using AcV5 (**G**) and AcV1 (**H**) antibodies. Mutant virus-infected cells were fixed with 4% paraformaldehyde at 48 h p.i. and subjected to a cELISA using AcV5 and AcV1 antibodies. AcV1 binding signals of mutant viruses were normalized to their AcV5 binding signals at pH 6.2. Error bars represent the SD from three independent replicates (*n *= 3).

To further examine the pH sensing characteristics of these mutants, we performed a cELISA using AcV5 and AcV1 antibodies (3). AcV5 targets a nine-amino acid epitope (residues 431–439) near the C-terminus of AcMNPV GP64, whereas AcV1 selectively binds the prefusion conformation of the basic loop (BL) but not its postfusion form (12). Our data showed that low-pH-induced conformational changes did not alter AcV5 binding (Fig. 2A). When AcV1 binding was normalized to WT GP64 (using AcV5 binding at pH 6.2 as a reference), the mutants fell into distinct categories. Category I: Mutants H194A, H221A, and H261A displayed markedly reduced AcV1 binding, indicating that these mutations allow a conformational transition similar to WT upon acidification (Fig. 2B). Category II: Moderate binding reduction (9%–17%) was seen for H394A and H446A. Category III: Pronounced binding reduction (34%–45%) was observed for H15A, H32A, and H39A. Category IV: Mutants H168A, H172A, H320A, H340A, H342A, and H351A exhibited flat binding curves across pH levels, suggesting they adopt a stable, postfusion-like BL conformation that is unresponsive to further pH-induced changes.

Although MFP fusogenicity is essential for virus infectivity, BmNPV employs dual fusion mechanisms (13), meaning that GP64’s activity is not strictly essential. To assess infectivity, each mutant was reintroduced into *gp64*-null bacmid (BmBac^Δgp64^) and evaluated via transfection and budded virus (BV) titration. Notably, mutants H168A, H172A, H342, and H351 failed to generate progeny BV, whereas other mutants produced varying BV production levels (Fig. 2C). Interestingly, despite unchanged AcV1 binding profiles upon acidification, both H320A and H340A retained infectivity. Furthermore, although H39A appeared non-fusogenic in transient assays, the recombinant bacmid bearing this mutation maintained fusion capacity and infectivity, possibly reflecting the cholesterol-independent fusion pathway of BmNPV (11, 13). Neutralization with the AcV1 antibody inhibited mutant infection to varying degrees (Fig. 2D), suggesting that AcV1 binding interferes with the conformational changes required for efficient infectivity, particularly in mutants H320A and H340A, which show stable AcV1 binding during the transition.

We further analyzed GP64 mutant conformational changes in virus-infected cells. Viruses H320A, H394A, and H446A were chosen based on their potential impact on pH sensing and fusogenicity. BmN cells were infected with these viruses at a multiplicity of infection (MOI) of 1 TCID_50_, and both cell surface expression and fusogenicity were assessed at 48 h post-infection (h p.i.) via cELISA and fusion assays. Cell surface expression levels were comparable among WT, H320A, H394A, and H446A viruses (Fig. 2E). However, H320A- and H446A-infected cells exhibited significantly reduced fusogenicity compared to WT, consistent with transfection-based assays (Fig. 2F). Furthermore, cELISA analysis using AcV5 and AcV1 antibodies under various pH conditions showed that AcV5 binding remained stable across all mutants (Fig. 2G). While H394A and H446A exhibited AcV1 binding curves similar to WT, the H320A mutant consistently displayed lower AcV1 binding (Fig. 2H), mirroring the pattern observed in transient expression assays (Fig. 2B). This indicates that the reduced fusogenicity and AcV1 binding of these mutants are not due to differences in expression levels.

Our comprehensive analysis identified H168, H172, H342, and H351 as key modulators of GP64-mediated membrane fusion. The localization of H168 and H172 within fusion loop 2 (a region critical for membrane interaction) and the complete loss of fusion and infectivity in their respective mutants underscore their indispensable roles. Previous studies have demonstrated that mutations spanning residue aa168–172 abolish fusogenicity (7, 13), notably, the substitution of histidine (H155 in AcMNPV GP64) with tyrosine at position 171 (Y171) in BmNPV GP64 is crucial for membrane interaction and may represent an adaptive modification for *Bombyx mori* cell infection (7, 14). In contrast, mutations in F169 and A170 do not affect GP64 conformational changes in virus-infected BmN cells (13), and the corresponding sites in AcMNPV GP64 are not involved in membrane interactions (15). Moreover, our observations that H168A and H172A mutants do not undergo conformational changes upon pH induction further support that their complete loss of fusogenicity and BV production results from a specific defect in pH sensing rather than altered receptor binding.

In addition, mutations within helix B (H320, H340, H342, and H351) significantly reduced BL-AcV1 binding, indicating that alterations in this region may affect epitope structure. Although H320A and H340A mutants retained fusogenicity and infectivity, the loss of these functions in H342A and H351A underscores the essential roles of these residues in membrane fusion. Importantly, unlike in AcMNPV GP64 (5), these residues appear dispensable for GP64 trimerization in BmNPV, suggesting a structural divergence in helix B between these viruses. Together, our findings support the notion that H342 and H351 serve as critical pH sensors that trigger GP64-mediated fusion activation.

In summary, our data indicate that H168, H172, H342, and H351 function as the principal pH sensors in BmNPV GP64-mediated fusion. Future structural investigation of BmNPV GP64 will elucidate the precise molecular interactions underlying its unique pH-dependent activation. This work not only advances our understanding of viral fusion activation and host adaptation in BmNPV but also provides insights applicable to other enveloped viruses.
